# The clinical course and outcomes following arthroscopic frozen shoulder 360° release

**DOI:** 10.1016/j.jseint.2024.07.006

**Published:** 2024-07-24

**Authors:** Brandon Ziegenfuss, Kristine Italia, Kathir Azhagan Stalin, Sarah Whitehouse, Ashish Gupta, Kenneth Cutbush

**Affiliations:** a Queensland Unit for Advanced Shoulder Research (QUASR), Queensland University of Technology, Brisbane, Australia; b St. Luke’s Medical Center, Manila, Philippines; c School of Mechanical, Medical and Process Engineering, Queensland University of Technology, Brisbane, Australia; d Greenslopes Private Hospital, Brisbane, Australia; e School of Medicine, The University of Queensland, Brisbane, Australia

**Keywords:** Shoulder release, Arthroscopic, Frozen shoulder, Adhesive capsulitis, Range of motion, 360° release

## Abstract

**Background:**

Frozen shoulder (FS) is a debilitating inflammatory condition affecting the shoulder capsule that causes significant pain and stiffness. Its etiology, pathophysiology, and treatment remain poorly understood. Although regarded as self-limiting, FS can have profound implications on the activities of daily living and usually takes 1-4 years to resolve on its own accord. In recalcitrant or severe cases where active range of motion (AROM) is extensively restricted, an arthroscopic 360° release may be performed. The aim of this study is to evaluate the clinical outcomes following the FS 360° arthroscopic release.

**Methods:**

An observational prospective cohort study was conducted assessing patient-reported outcome measures (PROMs) in patients who underwent the 360° arthroscopic release between July 2013 and January 2019. Various questionnaires were used to evaluate their shoulder preoperatively and at 2 weeks, 6 weeks, 3 months, 6 months, 12 months, and 24 months postoperatively. Relevant PROMs included the Oxford Shoulder Score; Western Ontario Shoulder Instability Index; Disabilities of the Arm, Shoulder, and Hand; Constant-Murley Score; American Shoulder and Elbow Surgeons score; and general measures of pain intensity (visual analog scale) and well-being (EQ-5D-3L). AROM movements included forward flexion, abduction, external rotation, internal rotation, as well as external and internal rotation at 90° of abduction.

**Results:**

Fifty consented patients underwent the arthroscopic FS 360° release. The mean age was 52.1 ± 7.7 years (range 35-72), and mean body mass index was 27.1 ± 4.7 kg/m^2^ (range 19.5-37.5). All PROMs, AROM movements, patient satisfaction, and EQ-5D-3L scores improved significantly between preoperative and 24-month time points (*P* < .001). Within-participant analysis demonstrated that there was no significant difference between the pathological shoulder AROM and the contralateral (healthy) shoulder AROM (collected preoperatively) for any movement at 24 months postoperatively (all *P* > .05). No complications or reoperations were reported.

**Conclusion:**

The arthroscopic 360° release is an effective and safe treatment modality for severe or recalcitrant FS. Statistically and clinically significant improvements in AROM and PROMs (Oxford Shoulder Score; Western Ontario Shoulder Instability Index; Disabilities of the Arm, Shoulder, and Hand; Constant-Murley Score; and American Shoulder and Elbow Surgeons score) occurred shortly after the surgery and progressively improved from 2 weeks to 24 months postoperatively, with the operative shoulder achieving similar range of motion as the nonpathological contralateral shoulder at 24 months.

The shoulder joint stands out as the most mobile joint in the human body, with its anatomy and kinematics affording it an extensive range of motion (ROM). Adhesive capsulitis (or “frozen shoulder” [FS]) is a common condition affecting shoulder mobility and causes considerable and prolonged pain and stiffness. The symptoms are debilitating, contributing to sleep disturbance and limiting activities of daily living including self-care.^46^ Despite its relatively high prevalence, affecting up to 5.3% of the population, FS remains inadequately defined,^17^ with its pathogenesis poorly understood and a conspicuous absence of consistent criteria for its diagnosis and treatment.^27^^,^^35^^,^^46^^,^^55^

FS is characterized by a fibrotic, inflammatory contracture primarily affecting the joint capsule.^40^ Advanced diagnostic techniques, including arthroscopy and microbiological analyses, have identified the rotator interval, long head of biceps tendon, coracohumeral ligament, and the subacromial bursa as additional structures associated with the pathogenesis of the condition.^46^ Recent histological investigations have further reported the presence of inflammatory biomarkers within the associated tissues including cytokines such as tumor necrosis factor α, interleukin 6, and interleukin 1 α and β.^7^^,^^36^^,^^46^

In 1975, Reeves discussed the natural history of FS and identified 3 disease stages: the painful phase (10-36 weeks), a period of stiffness (4-12 months), and a gradual recovery where a spontaneous return to ROM is seen (5-26 months).^8^^,^^42^ Despite the chronicity of symptoms and disability, FS is widely regarded as a self-limiting condition that is often initially managed conservatively and symptomatically.^1^^,^^34^ Common modalities of treatment include oral pain and anti-inflammatory medications, physical therapy, and intra-articular injections of cortisone with local anesthetic.^19^^,^^34^^,^^51^

However, several studies on the natural history of FS have shown that complete resolution was not achieved in all cases.^22^^,^^28^^,^^42^^,^^47^^,^^54^ Shaffer et al (1992) revealed that at 7 years, 50% of cases experienced mild pain and 60% still demonstrated some restriction of motion.^47^ A study by Hand et al (2008) involving 269 shoulders reported that at 4.4 years from the onset of symptoms, 41% still reported mild-to-moderate symptoms, while 6% still had severe ongoing symptoms with pain and functional loss.^22^ A systematic review by Wong et al (2017) involving 239 shoulders with idiopathic FS revealed that while ROM improved, its range remained outside the bounds of ‘normal’ and therefore concluded that full recovery of function was not consistently apparent.^54^ Moreover, Reeves (1975), who proposed that FS was self-limiting, showed in his original article that only 39% of patients had fully recovered.^42^ Finally, a recent study reported persistent limitation of motion in 39.7% of patients and residual pain with severity of visual analog scale (VAS) ≥ 3 after 2 years in 20.1% of patients following conservative treatment.^28^ Surgical management is believed to be of benefit for this group of patients, particularly those who inadequately respond to conservative management and who continue to experience residual shoulder pain and stiffness.^18^

Arthroscopic capsular release is a surgical option for patients who have debilitating limited ROM. A 360° full-thickness release of the shoulder capsule, including all the involved capsular tissue, has been reported in literature.^11^ This technique is indicated in patients presenting with severe stiffness (eg, external rotation of <30^o^), where the shoulder had failed to spontaneously recover or in patients who are unable to wait for the shoulder to resolve on its own accord.^11^ This study investigates the clinical outcomes of the 360° arthroscopic circumferential release procedure for severe or recalcitrant FS, and includes a comparison of outcomes between primary and secondary FS cohorts.

## Materials and methods

### Study design

This is a single-center, single-surgeon, prospective observational cohort study. Written and informed consent was obtained from patients who underwent an arthroscopic FS 360° release between July 2013 and January 2019. Participants were aged 18 years or more and diagnosed with either idiopathic or secondary post-traumatic FS. This study was approved by the Institutional Review Board of Brisbane Private Hospital (LREC/15/BPH/4).

### Participants

Indications for surgery were debilitating functional restriction due to capsular contracture resulting in severely limited active and passive external rotation (<30° at 0° abduction), and previous failed conservative management (including physiotherapy and/or cortisone injections). All surgeries were performed by a fellowship-trained shoulder surgeon (K. C.). Patients were excluded if they developed shoulder stiffness following previous surgery, had an open shoulder release procedure, or were diagnosed with concomitant shoulder pathologies (eg, rotator cuff tear), musculoskeletal injuries, and/or neurovascular disorders of the ipsilateral shoulder resulting in muscle weakness or loss of function. Patients with pathology occurring simultaneously on the contralateral shoulder were also excluded. Moreover, those who were pregnant, presented with other medical conditions such as central nervous system disturbance, alcohol or drug addiction that would impact clinical evaluation, had high medical dependency making assessment difficult, or were unwilling or unable to comply with the study protocol were also excluded.

### Surgical technique

**Figure 1:**
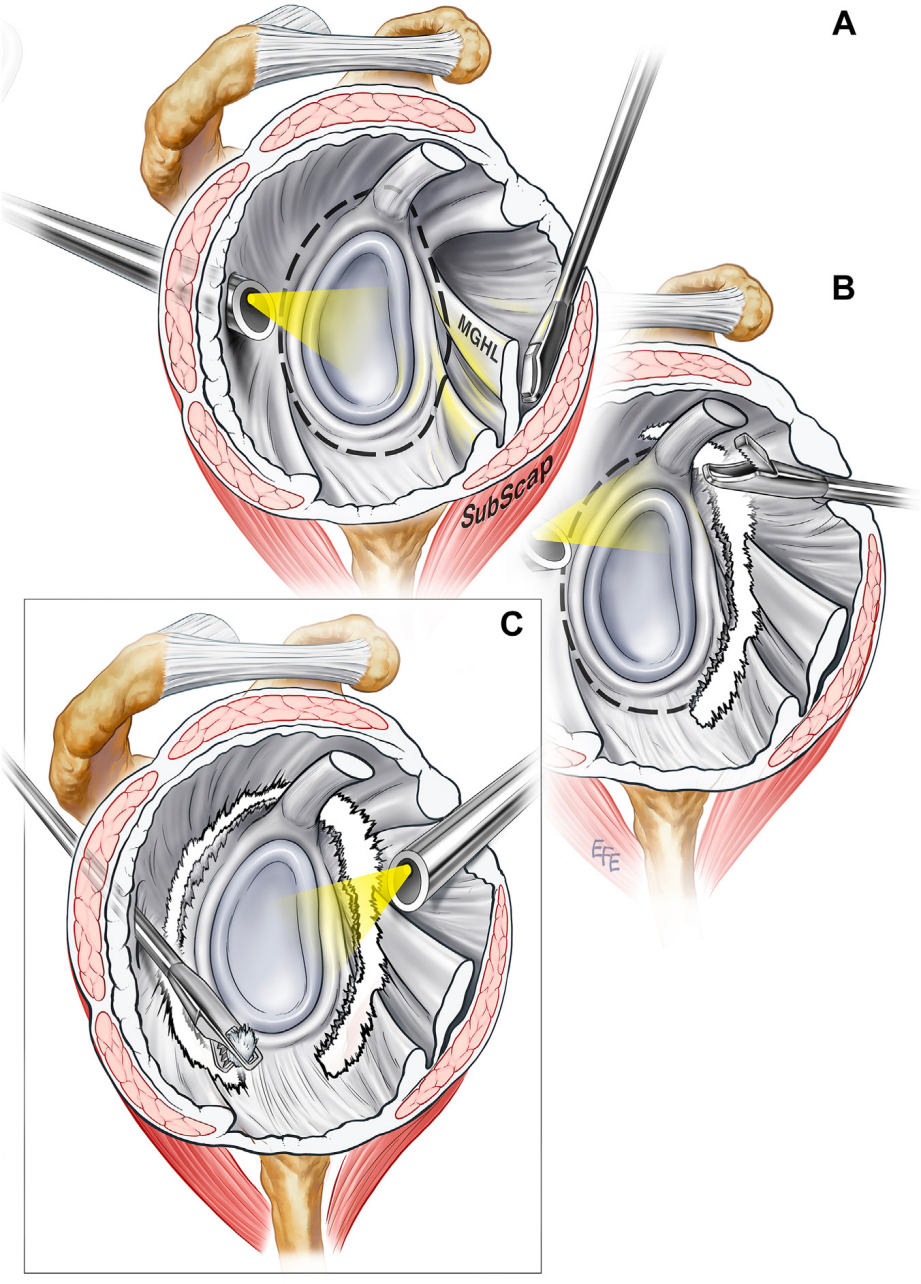
A lateral view of an illustrated right shoulder undergoing the 360° full thickness arthroscopic release technique. (**A**) The arthroscope is placed in the posterior portal while the anterior portal is established immediately lateral to the coracoid tip using an outside-in technique. (**B**) Viewing posteriorly, the anterior capsular release is commenced with the straight punch. The capsule is circumferentially released in an anticlockwise manner until the punch reaches the posterior portal. (**C**) The remaining posterior and inferior capsular release is completed while viewing is conducted from the anterior portal.

The circumferential FS release technique is described by Cutbush et al^11^ (Fig. 1). After general anesthesia is given, examination under anesthesia is performed to confirm the limitation of motion. The patient is then placed in a lateral decubitus position with the arm placed in 30° abduction and 10° forward flexion with lateral distraction of the glenohumeral joint using a 3-Point Shoulder Distraction System (AR- 1600 M; Arthrex, Naples, FL, USA) and traction through the hand using an Atraumatic Hand Holder Traction Attachment (AR-1602D; Arthrex, Naples, FL, USA). A standard posterior portal placed 2 cm inferior and medial to the posterolateral corner of the acromion is created and used as a viewing portal. Diagnostic arthroscopy is done using a 4-mm 30° arthroscope (Stryker, Kalamazoo, MI, USA). The diagnosis of FS is further confirmed by the presence of capsulitis and fibrous capsular thickening. If it is impossible or difficult to access the glenohumeral joint due to severe contracture, entry into the glenohumeral joint may be achieved through the rotator interval with the arthroscope placed in the anterior subacromial space.

Once diagnostic arthroscopy of the glenohumeral joint is complete, an anterior working portal is established using a 14-gauge needle inserted immediately lateral to the coracoid tip and through the rotator interval. A soft tissue chondrotome (FMS Tornado Micro Shaver; DePuy Mitek, Raynham, MA, USA) is introduced through the anterior portal and is used to excise the rotator interval. Capsular release then follows. A straight arthroscopic punch (Capsular Release Punch; ACUFEX; Smith & Nephew, Andover, MA, USA) is introduced through the anterior portal and pushed between the posterior aspect of the subscapularis muscle and the middle glenohumeral ligament to create a plane (Fig. 1, *A*). Once this plane is created, the punch is withdrawn to the level of the superior margin of the subscapularis tendon and then advanced to progressively divide the middle glenohumeral ligament, anterior capsule, and anterior band of the inferior glenohumeral ligament immediately adjacent to the labrum until the 6 o’clock position is reached. A punch with a 30° upward angle (Capsular Release Punch; Up-swept; ACUFEX; Smith & Nephew, Andover, MA, USA) can then be used to release the capsule past the 6 o’clock position. Once the limit of this inferior release is reached, the straight punch is used again to release the superior capsule immediately superior to the insertion of the long head of the biceps tendon and adjacent to the glenoid margin (Fig. 1, *B*). A soft tissue chondrotome or electrocautery (VAPR COOLPULSE 90 Electrode; DePuy Mitek, Raynham, MA, USA) may also be used for this part of the release. The release is extended until the arthroscope is reached in the posterior capsule. The arthroscope is then transferred to the anterior portal and the straight punch is introduced through the posterior portal. Any remaining intact capsule between the posterior portal and the superior capsule is divided. The posterior capsule, posterior band of the inferior glenohumeral ligament, and the remaining inferior capsule are then divided until the anterior inferior capsular incision is joined (Fig. 1, *C*). Full-thickness capsular release is confirmed by visualizing muscle fibers in the base of the incision throughout the circumferential capsular incision.

### Postoperative physiotherapy

Postoperative rehabilitation guidelines are divided into 3 phases. During Phase 1 (0-2 weeks postoperatively), physiotherapy for ROM exercises is started on the afternoon of the day of the surgery and the following morning prior to discharge. The patient is thereafter seen as an outpatient 2-3 times a week. Prescribed exercises are performed 4 times a day, although heavy lifting above shoulder height activities ought to be avoided as they may evoke a painful stimulus. To maintain adequate passive external rotation, a shoulder bolster is used when resting and at night. During Phase 2 (2-6 weeks postoperatively), the patient is seen 1-2 times a week with a goal of maintaining passive ROM gained in surgery to prevent adhesions. The patient is encouraged to use the arm for light to moderate activities. Finally, during Phase 3 (week 6+ postoperatively), the goal is to achieve a return to normal function and strength. To do this, patients are seen as required, with progressive rotator cuff and upper limb strengthening exercises applied.

### Demographic and functional outcome measures

Demographic information was collected preoperatively including age, sex, height, weight, hand dominance, affected shoulder side, occupation, concomitant medical conditions such as diabetes mellitus or thyroid disorders, and history of the presenting condition including the onset of the symptoms prior to the surgery. Other calculated demographic variables collected include body mass index and Charlson Comorbidity Index (CCI).^9^ The CCI quantifies the burden of disease and corresponding 1-year mortality risk based on 12 comorbidities. The maximum CCI score is 24 points, with a higher CCI indicating a higher risk of death.^9^

All patients underwent preoperative assessment comprising of shoulder ROM tests of both shoulders and patient-reported outcome measures (PROMs) (all on a worst to best scale unless otherwise specified). ROM assessment included forward flexion, abduction, external and internal rotation at 0° abduction, and external and internal rotation at 90° abduction. Shoulder function was assessed using the Oxford Shoulder Score^14^ (OSS) (possible range: 0-48, minimal clinically important difference [MCID]: 5.1^13^), the American Shoulder and Elbow Surgeons^43^ (ASES) (range: 0-100, MCID: 6.4^13^), and the Constant-Murley Score^10^^,^^53^ (CMS) (range: 0-100, MCID: 8.0^13^). Symptoms were assessed using the shortened version of the Disabilities of the Arm, Shoulder, and Hand (QuickDASH) Short Form^24^ (range: 0-100, MCID: 8.0^13^) and a VAS (range: 0-10 [pain scores are measured as no pain to worst pain], MCID: 1.37^13^). Shoulder stability was assessed using the Western Ontario Shoulder Instability Index^29^ (WOSI) (range: 0-2100, MCID: 60.7^13^). MCID values were also compared with thresholds recently published by Pasqualini et al^41^ with similar values of 8.2 for ASES, 10.1 for CMS, and 1.1 for VAS. Patient’s quality of life was assessed using the Euro Quality of Life Measure^6^^,^^16^ (EQ-5D-3L, range: 0-1, MCID: 0.0485^26^). Assessments of shoulder ROM and PROMs were repeated at 2 weeks, 6 weeks, 3 months, 6 months, 12 months, and 24 months postoperatively. Complications noted at any time point following surgery were recorded.

### Statistical analysis

IBM SPSS statistical software (version 26; IBM Corp., Armonk, NY, USA) was used for statistical analysis. The sample size was chosen to enable a minimum follow-up period of 2 years, with a reasonable number of cases from inception of this new technique to identify safety based on clinical experience. Baseline demographic data were analyzed with means, standard deviations, and range for continuous variables and frequencies for categorical variables. Paired sample *t*-tests were used to analyze the difference between preoperative and postoperative scores for each outcome variable. Significance testing was performed using analysis of variance or the Mann-Whitney U test as appropriate to the data type. Frequencies were compared using the chi-squared test or Kendall’s tau-c for ordinal responses. No adjustments were made for multiple testing. For all analyses, *P* ≤ .05 was considered statistically significant.

## Results

### Patient demographic characteristics

A total of 53 arthroscopic FS 360° release procedures were performed between July 2013 and January 2019 (Table I). Two patients were excluded as they declined participation. One patient developed FS in both shoulders; however, the symptoms in the contralateral shoulder only started 22 months after the onset in the initial shoulder. The mean age was 52.1 ± 7.7 years (range 35-72) and mean body mass index was 27.1 ± 4.7 kg/m^2^ (range 19.5-37.5). More than half of the patients were females (58.8%), with the dominant side being affected in 19 (37.3%) patients. Forty (78.4%) were employed or currently on sick leave, and of these individuals, 40% engaged in a manual occupation. In terms of comorbidities, 17 patients had a Charlson Comorbidity Score (CCS) of 0, 23 patients had a CCS of 1, 9 patients had a CCS of 2, and 2 patients had a CCS of 4. Among the 50 patients, 6 (11.8%) patients were smokers, 7 (13.7%) had diabetes mellitus, and 6 (11.8%) had a thyroid disorder.

**Table I:** Population demographic variables.

Age (mean, SD, range)	52.1, 7.7, 35-72
Sex	
Male	21 (41.2%)
Female	30 (58.8%)
BMI (mean, SD)	27.1, 4.7
Pathological side	
Dominant	19 (37.3%)
Nondominant	32 (62.7%)
Occupation status	
Employed	40 (78.4%)
Unemployed	3 (5.9%)
Retired	8 (15.7%)
Occupation type	
Manual	13 (25.5%)
Office	24 (47.1%)
Manual and office	3 (5.9%)
Unemployed	3 (5.9%)
Retired	8 (15.7%)
Smoking status	
Smoker	6 (11.8%)
Nonsmoker	45 (88.2%)
Comorbidities	
Diabetes mellitus (I or II)	7 (13.7%)
Thyroid disorders (undefined)	6 (11.8%)
Charlson Comorbidity Index	
0	17 (33.3%)
1	23 (45.1%)
2	9 (17.6%)
3	0
4	2 (3.9%)

*SD*, standard deviation; *BMI*, body mass index.

### Pathology characteristics

FS was diagnosed as primary in 43 (84.3%) patients, and as secondary in 8 (15.7%) patients (Table II). Review of history revealed presence of trauma (fracture, soft tissue injury, and dislocation) to the affected shoulder in 21 patients (41.2%). Thirty-six patients (70.6%) described experiencing symptoms dating less than or equal to 12 months prior to surgery, while 15 (29.4%) patients were symptomatic for more than 12 months (range 0-3 months to >36 months). Preoperatively, 36 (76.6%) patients had at least one cortisone injection (subacromial or glenohumeral joint) before the decision to undergo surgery was made. Seventeen patients received 1 injection, 14 received 2 injections, and 5 patients received 3 injections. Seventeen patients (33.3%) underwent physiotherapy preoperatively. Eighteen (35.3%) had a previous upper limb surgical procedure, while 33 did not have a history of upper limb surgery. All patients underwent physiotherapy postoperatively as per the physiotherapy guidelines.

**Table II:** Pathology characteristics, diagnosis, and preoperative/prior treatments.

Diagnosis	
Primary	43 (84.3%)
Secondary	8 (15.7%)
Primary diagnosis	
Trauma	21 (41.2%)
Other	30 (58.8%)
Symptomatic time period	
0-12 mo	36 (70.6%)
>12 mo	15 (29.4%)
Treatment prior to surgery	
Physiotherapy	17 (33.3%)
Total injection	
0	11 (21.6%)
1	17 (33.3%)
2	14 (27.5%)
3	5 (9.8%)
Previous upper limb surgery	
Yes	18 (35.3%)
No	33 (64.7%)

### Functional outcomes

**Figure 2:**
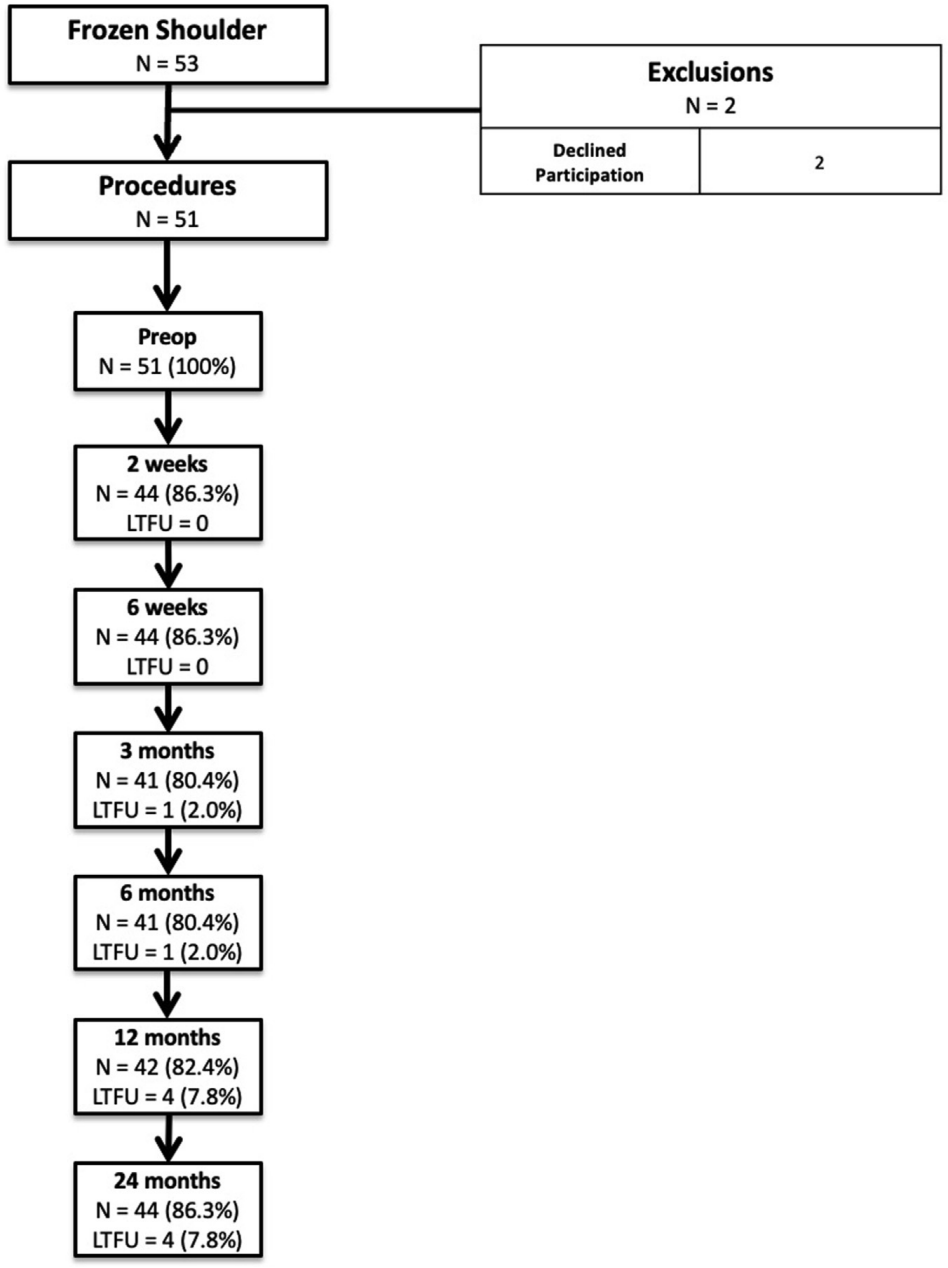
Inclusion flowchart. *LTFU*, lost to follow-up.

Data were collected preoperatively (100%), and at 2 weeks (follow-up: 86.3%), 6 weeks (follow-up: 86.3%), 3 months (follow-up: 80.4%), 6 months (follow-up: 80.4%), 12 months (follow-up: 82.4%), and 24 months (follow-up: 86.3%) postoperatively. Follow-up and loss to follow-up (LTFU) rates are demonstrated in Figure 2. LTFU is herein defined as the postoperative time point from which a patient’s clinical assessment and all subsequent time point assessments were missed.

### Range of motion

**Figure 3:**
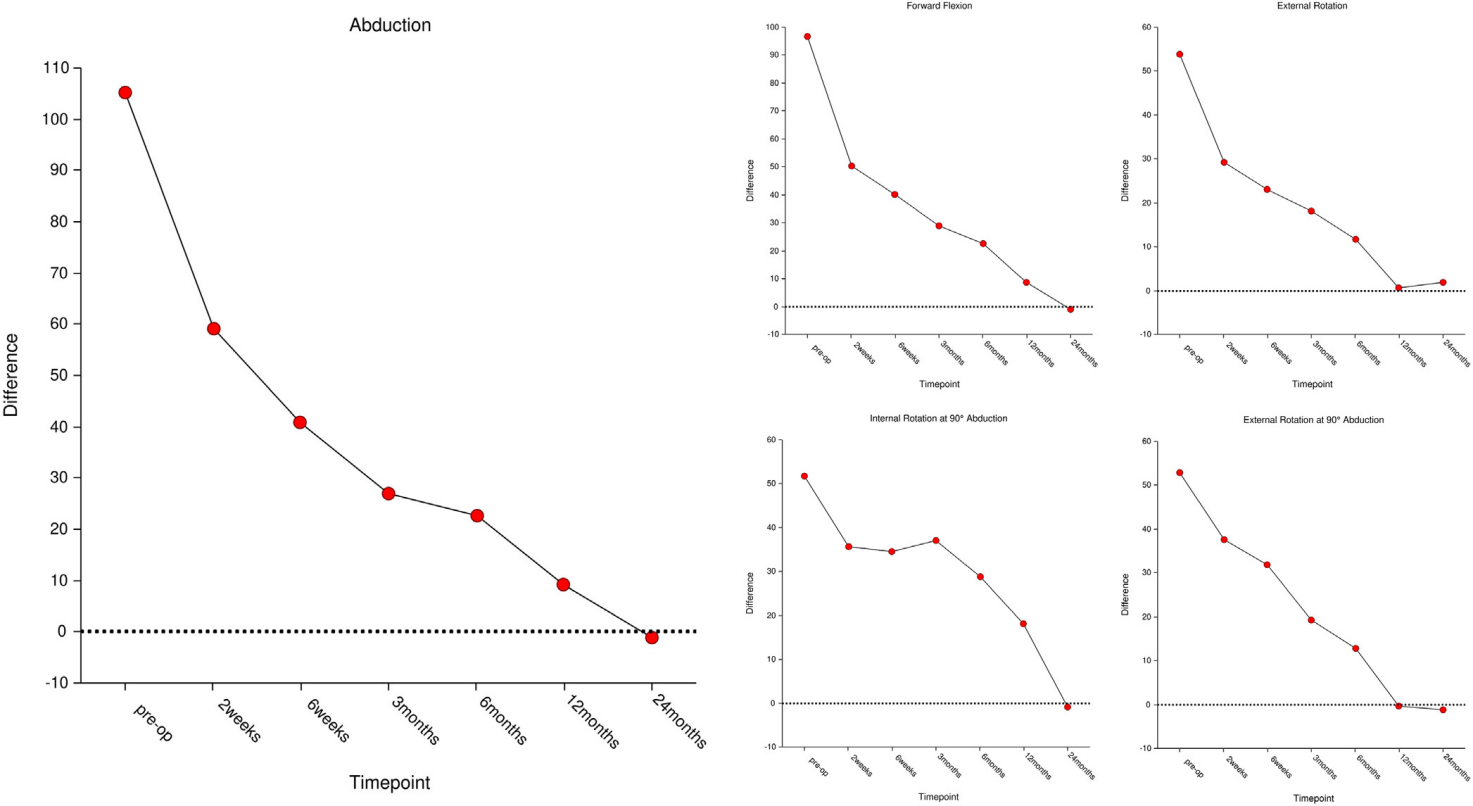
Mean within participant changes in AROM between pathological side and healthy nonoperative sides. These graphs indicate the average patient-specific improvement relative to their optimal ROM with the horizontal dotted line indicating zero difference. *AROM*, active range of motion; *ROM*, range of motion.

Table III demonstrates the mean active ROM (AROM) movements (forward flexion, abduction, external rotation, and internal rotation at 0° and 90° of abduction) of the operative arm at each time point, as well as nonpathological arm AROM prior to the operative intervention. There was a continuous and statistically significant improvement in abduction, forward flexion, internal rotation, and external rotation from 2 weeks post operatively until 24 months post-operatively (Fig. 3). A small decline was noted in external rotation at 90° of abduction from 12 months to 24 months, and in internal rotation at 90° of abduction from 2 weeks to 3 months; however, these were not statistically significant (*P* = .648 and *P* = .180, respectively). Paired *t*-test analysis revealed significant improvements in all AROM movements between preoperative and 24-month time points (*P* < .001). Importantly, no significant differences between operative arm AROM and the nonpathological arm AROM were seen at 24 months postoperation (*P* > .05) (Fig. 3). There were no statistically significant differences found across all AROM movements between primary and secondary FS cohorts.

**Table III:** Operative arm and nonoperative arm (NOP) active ROM (AROM) reported as mean + - SD with sample sizes listed (n).

OP AROM	Preoperative (NOP)	Preoperative	2 weeks	6 weeks	3 mo	6 mo	12 mo	24 mo	Preoperative vs. 24 mo
Forward flexion	170.3 ± 14.2, 46, 130-180	75.1 ± 31.5, 50, 9-145	121.5 ± 25.3, 43, 70-180	132.0 ± 20.9, 43, 80-180	141.7 ± 21.8, 38, 80-180	148.6 ± 16.7, 39, 120-180	163.0 ± 14.6, 37, 135-180	171.3 ± 10.6, 40, 140-180	−17.027∗ (*P* < .001)
Abduction	170.1 ± 21.7, 46, 70-180	64.0 ± 26.9, 48, 0-150	114.3 ± 38.8, 42, 20-180	131.2 ± 27.8, 43, 70-170	141.5 ± 33.2, 38, 50-180	147.0 ± 32.6, 39, 20-180	163.4 ± 16.5, 37, 110-180	170.5 ± 12.9, 40, 140-180	−21.063∗ (*P* < .001)
External rotation	69.2 ± 19.0, 46, 10-90	15.7 ± 15.5, 50, −15 to 50	40.2 ± 14.7, 43, 10-70	45.9 ± 14.4, 43, 0-75	49.7 ± 14.6, 38, 10-80	56.9 ± 14.5, 39, 10-80	65.0 ± 17.2, 37, 10-95	67.0 ± 16.9, 40, 10-90	−15.311∗ (*P* < .001)
Internal rotation									
Lateral thigh	0	9 (18.4%)	3 (7.3%)	0	1 (2.6%)	0	0	0	0.133† (*P* = .235)
Buttock	0	34 (69.4%)	6 (14.6%)	0	0	1 (2.6%)	1 (2.7%)	0	
LS junction	2 (3.9%)	3 (6.1%)	7 (17.1%)	7 (16.3%)	2 (5.3%)	0	1 (2.7%)	3 (7.5%)	
L3	7 (13.7%)	1 (2.0%)	5 (12.2%)	11 (25.6%)	5 (13.2%)	4 (10.3%)	4 (10.8%)	5 (12.5%)	
T12	11 (21.6%)	2 (4.1%)	17 (41.5%)	22 (51.2%)	21 (55.3%)	14 (35.9%)	13 (35.1%)	12 (30.0%)	
T7	25 (49.0%)	0	3 (7.3%)	3 (7.0%)	9 (23.7%)	20 (51.3%)	18 (48.6%)	20 (50.0%)	
ER90	83.7 ± 15.8, 46, 10-110	20.5 ± 20.4, 16, 0-55	46.7 ± 25.2, 39, −20 to 90	51.9 ± 25.3, 42, -25 to 85	63.2 ± 21.9, 38, 0-90	70.9 ± 15.6, 39, 30-100	83.2 ± 8.7, 37, 60-95	82.9 ± 10.3, 40, 45-90	−7.938∗ (*P* < .001)
IR90	70.8 ± 22.1, 46, −20 to 90	12.1 ± 15.8, 17, 0-50	39.3 ± 19.5, 38, 0-100	38.5 ± 24.6, 42, −60 to 80	33.0 ± 19.8, 38, 0-80	43.6 ± 17.8, 39, 0-70	56.9 ± 20.1, 37, 15-90	73.7 ± 14.3, 40, 40-90	−16.237∗ (*P* < .001)

*SD*, standard deviation; *ROM*, range of motion; *ER90*, external rotation at 90°; *IR90*, internal rotation at 90°.

External/internal rotation at 90° of abduction (ER90, IR90).

∗ T-test score.

† Kendall’s Tau Correlation. Significance denoted where *P* ≤ .05.

### Patient-reported outcomes scores

Table IV demonstrates the mean functional shoulder scores (OSS, WOSI, QuickDASH, ASES, and CMS), EQ-5D-3L, and VAS scores for pain and patient satisfaction at each time point. Paired *t*-test analysis revealed significant improvements for all functional outcome measures between preoperative and 24-month time points (*P* < .001). Additionally, significant improvements in VAS measurements between preoperative and 24-month time points assessing pain while undertaking normal activities of daily living, at rest, night, and at its worst were reported (*P* < .001). Overall satisfaction VAS and EQ-5D-3L scores also improved significantly between these time points (*P* < .001). There were no statistically significant differences between primary and secondary FS cohorts for the ASES and QuickDASH scores across all time points (*P* > .05). However, patients diagnosed with primary FS reported a significantly greater mean CMS compared to their secondary FS counterparts at 12 and 24 months postoperatively (82.7 vs. 71.7 and 84.3 vs. 73.0, both *P* = .045).

**Table IV:** Patient-reported outcome measures (PROMs) recorded preoperatively and at each postoperative time point (Mean ± SD, n, range).

PROM	Preoperative	2 weeks	6 weeks	3 mo	6 mo	12 mo	24 mo	Preoperative vs. 24 mo (T-score)
VAS (normal)	6.3 ± 2.3, 50, 1-10	3.9 ± 2.0, 40, 0-8	3.3 ± 2.1, 43, 0-7	2.3 ± 1.9, 41, 0-9	1.8 ± 1.9, 42, 0-7.6	0.9 ± 1.2, 42, 0-6	0.8 ± 1.6, 41, 0-6.5	14.528 (*P* < .001)
		82.1%	81%				100%	
VAS (pain at rest)	5.0 ± 2.8, 50, 0-10	2.6 ± 2.3, 40, 0-8	2.4 ± 2.2, 43, 0-9	1.4 ± 2.1, 41, 0-9	0.6 ± 1.0, 42, 0-3.5	0.5 ± 1.2, 42, 0-6	0.3 ± 0.7, 32, 0-3.5	8.807 (*P* < .001)
VAS (pain during night)	7.0 ± 2.1, 50, 2.3-10	4.3 ± 2.6, 40, 0-10	3.8 ± 2.6, 43, 0-10	3.0 ± 2.3, 41, 0-10	1.8 ± 2.0, 42, 0-8	1.0 ± 1.6, 42, 0-7	0.7 ± 1.3, 32, 0-5	16.844 (*P* < .001)
VAS (pain at its worst)	8.9 ± 1.2, 50, 5.8-10	6.4 ± 2.1, 40, 0-10	6.0 ± 2.6, 43, 1-10	4.7 ± 2.7, 41, 1-10	3.5 ± 3.0, 42, 0-10	2.1 ± 2.4, 42, 0-10	2.2 ± 3.1, 32, 0-10	12.875 (*P* < .001)
VAS (satisfaction with symptoms and function)	1.3 ± 1.9, 48, 0-8	7.2 ± 2.4, 40, 2-10	7.3 ± 1.9, 43, 3-10	7.6 ± 2.1, 41, 3-10	8.4 ± 2.0, 41, 2.5-10	9.2 ± 1.3, 42, 4-10	9.1 ± 2.0, 41, 0-10	−20.854 (*P* < .001)
VAS (shoulder appearance)	6.7 ± 3.3, 38, 0-10	8.9 ± 1.8, 36, 2-10	8.7 ± 1.9, 39, 4-10	8.9 ± 1.9, 38, 3-10	9.2 ± 1.3, 38, 5-10	9.2 ± 1.6, 41, 5-10	9.3 ± 1.9, 32, 0-10	−2.501 (*P* = .020)
OSS	18.5 ± 8.3, 51, 5-37	31.8 ± 7.1, 35, 18-46	35.2 ± 7.4, 40, 1.6-4.7	39.3 ± 7.2, 41, 1.1-4.7	41.6 ± 7.5, 42, 1.4-4.8	44.3 ± 4.4, 42, 3.4-4.8	45.3 ± 4.1, 41, 29-48	−19.011 (*P* < .001)
		82.9%	97.5%				100%	
WOSI	1574.0 ± 271.8, 38, 920-1990	1133.4 ± 402.8, 35, 100-1840	952.8 ± 407.6, 37, 190-1560	757.2 ± 431.7, 36, 30-1630	563.0 ± 427.7, 36, 0-1612	328.4 ± 334.1, 41, 0-1310	187.6 ± 284.6, 40, 0-1261	24.391 (*P* < .001)
		93.8%	97.1%				100%	
QuickDASH	61.4 ± 18.8, 50, 18.2-90.9	40.5 ± 17.5, 36, 2.3-79.6	33.5 ± 17.5, 40, 0-70.5	27.2 ± 19.6, 41, 0-88.6	17.6 ± 17.9, 42, 0-61.4	10.0 ± 12.6, 42, 0-45.5	7.3 ± 11.0, 40, 0-36.4	17.94 (*P* < .001)
		80.6%	87.5%				100%	
EQ-5D-5L	0.38 ± 0.35, 49, −0.2 to 1	0.62 ± 0.24, 36, −0.07 to 1	0.70 ± 0.21, 40, 0.06-1	0.72 ± 0.25, 41, −0.11 to 1	0.84 ± 0.22, 42, 0.29-1	0.90 ± 0.15, 42, 0.52-1	0.93 ± 0.13, 40, 0.52-1	−9.63 (*P* < .001)
		63.9%	70.0%				95%	
ASES	29.5 ± 16.0, 39, 1.7-58.3	54.9 ± 17.5, 33, 13.3-98.3	60.0 ± 19.5, 37, 25-100	70.5 ± 17.3, 37, 18.3-100	81.9 ± 17.6, 33, 40.4-100	89.6 ± 11.1, 40, 46.7-100	91.4 ± 13.5, 40, 44.2-100	−20.53 (*P* < .001)
		93.8%	91.7%				100%	
CMS	22.3 ± 12.0, 46, 2.5-47.7	48.5 ± 17.2, 34, 14.5-87.0	56.8 ± 13.6, 40, 25-84.9	64.7 ± 14.1, 38, 35.5-89.4	71.1 ± 15.7, 39, 33.5-98.9	81.0 ± 10.9, 33, 56.6-98.9	83.1 ± 10.4, 37, 50-100	−23.841 (*P* < .001)
		93.8%	97.4%				100%	

*SD*, standard deviation; *PROM*, patient-reported outcome measure; *VAS*, visual analog scale; *OSS*, Oxford Shoulder Score; *WOSI*, Western Ontario Shoulder Instability Index; *QuickDASH*, Disabilities of the Arm, Shoulder, and Hand; *ASES*, American Shoulder and Elbow Surgeons; *CMS*, Constant-Murley Score.

Proportion (%) of patients achieving the Minimal Clinically Important Difference (MCID) for OSS, ASES, CMS, QuickDASH, VAS (normal), WOSI, and EQ-5D-3L listed at 2 weeks, 6 weeks, and 24 months postoperation. Significance denoted where *P* < .05.

All patients reported clinically significant improvements for the OSS, ASES, CMS, QuickDASH, VAS, and WOSI by the 24-month postoperative time point, while 95% achieved the MCID for the EQ-5D-3L score by this time point. At 2 and 6 weeks postoperation, the majority of patients surpassed the MCID for the OSS (82.9%, 97.5%), ASES (93.8%, 91.7%), CMS (93.8%, 97.4%), QuickDASH (80.6%, 87.5%), VAS (normal; 82.1%, 81%), WOSI (93.8%, 97.1%), and EQ-5D-3L (63.9%, 70%), and this remained consistent when using the values recently published by Pasqualini.^41^

### Complications

There were no complications noted following all procedures. There were no cases of axillary nerve injury. There were no cases of gross dislocations observed, with low WOSI scores at each time point. There was one patient who presented with a sensation of instability at approximately 36 months after surgery. However, this patient was deemed hyperlax upon clinical assessment, and the symptoms required no further treatment. None of the patients had a recurrence of stiffness at a minimum of 2 years postsurgery.

## Discussion

Various treatment methodologies exist for the 3 stages in the natural history of FS. Research has indicated that while the condition is largely considered to undergo a spontaneous recovery, the symptomatic stages are largely unpredictable, highly impactful on the activities of daily living, and in some cases, do not progress to a spontaneous resolution. For this reason, a safe intervention that ensures fast, reliable, and consistent recovery from pain and stiffness is warranted.

A number of surgical interventions are used to treat FS. Manipulation under anesthesia (MUA) is a common procedure performed for patients suffering from FS with persistent limitation of motion.^2^^,^^21^^,^^37^ However, iatrogenic injuries following MUA have been reported, such as humeral/glenoid fractures, glenohumeral dislocation, rotator cuff tears, labral tears, hematoma, hemarthrosis, and brachial plexus injuries.^4^^,^^31^^,^^37^^,^^38^ The inherent difficulty in adequately controlling this procedure, along with its reported track record of severe complications, necessitates the use of an in-vivo surgical option.

The circumferential capsular release has been described in literature.^11^^,^^12^^,^^23^^,^^25^^,^^44^^,^^52^ Cutbush et al (2021) describe the arthroscopic 360° release technique to treat severe cases of FS.^11^ The procedure is indicated in cases where global AROM is restricted, and in particular, external rotation is limited to less than 30°.^11^ This study has investigated the patient clinical outcomes (pain, function, and wellbeing) following this procedure. Herein, we report that all measures of function (AROM, OSS, ASES, CMS, WOSI, and QuickDASH), pain and symptoms (QuickDASH and VAS), and patient general wellbeing (EQ-5D-3L) all significantly improved from preoperative to 24 months postoperatively (*P* < .001). At this time point, every patient reported improvements that exceeded the MCID across all PROMs (both functional and pain), deeming the improvements to be both statistically and clinically significant. Within-participant statistical analyses also revealed that after 24 months, there was no significant difference between the pathological arm AROM and the nonpathological arm AROM (*P* > .05 for all measures) (Fig. 3). Finally, comparisons between primary and secondary FS cohorts demonstrated no significant difference for AROM movements and ASES and QuickDASH scores at any time point. However, patients in the primary FS populations achieved a significantly greater CMS at 12 and 24 months postoperatively. While this is surprising, the differences in CMS ought to be interpreted with caution given relatively small patient population size in the secondary FS group (ie, n = 8). One need also consider the high likelihood of encountering a statistically significant difference in a study that has conducted a large number of analyses.

To the best of the author’s knowledge, this is the first study to clearly demonstrate the road to effective patient-specific AROM and PROMs recovery. Importantly, clinically significant improvements exceeding the MCID for each score were seen by the majority (up to 94%) of patients as early as 2 weeks postoperatively. QuickDASH experienced the lowest proportion of improvement at this point despite recording clinically significant improvements in 80.6% of patients. This highlights the success of the surgical intervention, and further supports its role in efficiently returning patient function and quickly improving symptoms in severe or recalcitrant FS patient populations (regardless of primary or secondary FS diagnosis).

Throughout this study, only one case presented with a sensation of instability (approximately 36 months postoperatively). However, this patient was deemed hyperlax upon clinical assessment and the symptoms required no further treatment. Therefore, no complications manifested from the complete capsular release in this study. In contrast to MUA, the 360° release technique allows a more controlled circumferential capsular release, avoiding the potential risks associated with MUA. Evidently, the successful return to function, AROM, and amelioration of pain with no added risk of complications show the technique by Cutbush et al (2021) to be both successful and safe.

These findings align with current literature exploring the clinical outcomes following capsule release.^3^^,^^12^^,^^18^^,^^33^^,^^49^ Cvetanovich et al (2018) reported that idiopathic cases of FS netted a significant improvement in their AROM as early as 2 weeks post complete capsule release.^12^ Moreover, they reported mean ASES and VAS (pain) outcomes of 97.0 and 0.2 at 24 months, respectively, with no complications or revision surgeries.^12^ Our mean ASES and VAS pain scores were 91.4 and 0.8, respectively, at the same time point. Similarly, Smith et al (2014) reported a mean OSS of 38.1 at 12 months post arthroscopic complete capsular release, while our study demonstrates an OSS of 44.3 at this time point.^49^ Le Lievre et al (2012) reported that outcomes (shoulder ROM, function, and pain) had significantly improved and were maintained and/or enhanced at 2 years postoperatively.^33^ Baums et al (2006) employed a partial release technique and demonstrated that at 12 months postoperatiely, patients reported a mean ASES score of 90, VAS score of 2, and AROM for abduction (150°), forward flexion (160°), and external rotation (60°) that is consistent with our findings at 12 months.^3^ Evidently surgical intervention nets patients markedly improved functional and symptomatic relief.

While some authors have advocated for a partial release by sparing the inferior capsule to avoid axillary nerve injury, by using an arthroscopic punch under direct vision, it is thought that a complete 360° capsule release can be achieved while safely preserving the axillary nerve.^11^ This technique, in combination with the employed lateral traction applied to the limb, improves visualization and lateral displacement of the axillary nerve, aiding its preservation.^11^ Joint instability following the circumferential capsule release also needs to be considered.^20^ Although present, this complication has not been widely reported in literature, with one case of frank dislocation that later also developed symptoms of shoulder instability.^20^ Importantly, no complications (including axillary nerve neuropathies and instability) were reported in this study’s population cohort.

The clinical outcomes of patients who have undergone conservative methods of treatment have been reported in literature.^19^^,^^32^^,^^39^^,^^45^ Common methods of treatment include oral medication, physiotherapy, hydrodilatation, intra-articular and subacromial corticosteroid injection therapy, acupuncture, and suprascapular nerve block. A randomized control trial by Russell et al (2014) concluded that hospital-based exercise classes result in rapid improvements in CMS, OSS, and AROM (forward flexion and external rotation), it being notably more effective than individual physiotherapy and home exercise programs.^45^ Importantly, the clinical outcomes reported in our study consistently outperform the improvements in CMS and AROM (forward flexion and external rotation) for every time point comparison (preoperative 6 weeks, preoperative 6 months, and preoperative 12 months) compared to those after physical therapy (Table V).

**Table V:** A comparison of changes in Constant-Murley Score (CMS) and active range of motion (forward flexion, external rotation) seen among baseline (including preoperative), 6 weeks postoperatively, 6 months postoperatively, and 12 months postoperatively for conservative^45^ and surgical interventions.

	Score (baseline)	Score 6 weeks	Delta	Score 6 mo	Delta	Score 12 mo	Delta
CMS							
Surgery	22.3	56.8	34.5	71.1	48.8	81	58.7
Exercise class	37.5	71.5	34	82	44.5	88.1	50.6
Individual physiotherapy	40.2	62.9	22.7	70.8	30.6	77.8	37.6
exercises	41.7	52	10.3	64.8	23.1	72	30.3
Forward flexion							
Surgery	75.1	132	56.9	148.6	73.5	163	87.9
Exercise class	95	140	45	153	58	166	71
Individual physiotherapy	96	136	40	151	55	165	69
Home exercises	96	112	16	129	33	146	50
External rotation							
Surgery	15.7	45.9	30.2	56.9	41.2	65	49.3
Exercise class	15	39	24	53	38	58	43
Individual physiotherapy	16	37	21	52	36	57	41
Home exercises	16	28	12	38	22	49	33

*CMS*, Constant-Murley Score.

The clinical outcomes following other conservative methodologies, such as cortisone injection and hydrodilatation (with or without cortisone injection therapy), have also been reported.^5^^,^^15^^,^^30^^,^^48^^,^^50^ Cortisone injection therapy is often used to treat adhesive capsulitis due to its cost-effectiveness and ease of administration. The progression to capsular fibrosis is prevented by controlling the synovitis present in the earlier stages of FS, and therefore, cortisone therapy is employed as an early treatment methodology.^15^^,^^30^ The use of cortisone injections has demonstrated short-term efficacy, with studies by Shah and Lewis (2007) reporting multiple (up to 3) corticosteroid injections resulted in improved ROM and pain for 6-16 weeks from the time of the first injection.^48^ These findings were consistent with Blanchard et al (2009). However, these authors reported that corticosteroid use had demonstrated inferior improvements in pain and ROM when compared to physiotherapeutic interventions in the longer term.^5^

Lädermann et al (2021) summarized meta-analyses of randomized controlled trials to compare various conservative FS treatment modalities.^32^ Their primary finding was that hydrodilatation with corticosteroid administration provided a significant pain relief and improved ROM in the short term at any given time point in the natural history of FS compared to standard corticosteroid use or physiotherapy.^32^ However, the procedure is often deemed painful (despite concurrent local anesthetic) and the treatment is largely considered as a short-term solution for an otherwise long-term pathology. For this reason, a successful and risk averse surgical option is indicated.

Although the results in this article provide a compelling argument for the use of the 360° capsule release in patients with severe FS, a number of limitations in this study exist. First, the population size and single-center nature of the study mean the applicability of the intervention to the general population ought to be carefully considered. The sample size (51 procedures) may be too small to detect any potential complications reported in literature (eg, instability, axillary nerve injury). Despite a relatively smaller sample size, the LTFU rate is considerably small (4) and our follow-up rates were consistently at or more than 80% throughout the follow-up period; however, there remains the possibility that those patients who were lost to follow-up were failures that sought treatment elsewhere.

Second, the results in this article pertain to a specific demographic and do not further assess the effect of additional potential prognostic influencing variables. Confounding variables include the existence of concomitant metabolic or autoimmune disorders (such as diabetes mellitus or thyroid disorders), preoperative therapy (including cortisone joint injections or physiotherapy), the length of the symptomatic time period prior to surgical intervention, as well as whether the FS is classified as idiopathic or secondary to injury. Further research is warranted to determine the PROMs following complete capsular release in these specific FS patient populations.

Finally, in treating FS, it is recommended that careful consideration of the patient’s experience of the disease and circumstance be made, as well as the stage of FS to effectively determine the appropriate course of medical or surgical intervention. Each treatment modality has its merits, benefits, and timing to effectively optimize the outcome for the patient.

## Conclusion

The arthroscopic 360° release is an effective and safe treatment modality for severe or recalcitrant FS. Statistically and clinically significant improvements in AROM; PROMs (OSS, WOSI, Quick-DASH, CMS, ASES, and EQ-5D-3L); and patient self-reported measures of pain, function, satisfaction, and wellbeing occurred shortly after the surgery and progressively improved from 2 weeks to 24 months postoperatively, with the operative shoulder achieving similar ROM as the contralateral normal shoulder at 24 months. Importantly, no complications were reported in this cohort throughout the course of the study.

## Acknowledgments

The authors would like to thank Ms. F. Stephenson and Mr. T. Shuker for their assistance in data collection. They also thank Dr. E. Levent for the artistic images.

## Disclaimers:

Funding: Kenneth Cutbush and Ashish Gupta received funding from the 10.13039/501100000923Australian Research Council through the Industrial Transformation Training Centre for Joint Biomechanics (IC190100020) and its associated industry partners. Cash-contributing partners include Stryker, Zimmer Biomet, Logemas, and Australian Biotechnologies. None of these funding sources were involved in data collection, data analysis, or the preparation of or editing of the manuscript.

Conflicts of interest: Kenneth Cutbush receives fellowship program support from Device Technology, Zimmer Biomet, Johnson & Johnson, and Wright Medical. Kenneth Cutbush receives research support from Zimmer Biomet, Wright Medical, and Ausbiotech. The other authors, their immediate families, and any research foundation with which they are affiliated have not received any financial payments or other benefits from any commercial entity related to the subject of this article.
